# Etoposide in patients with rheuma-associated hemophagocytic lymphohistiocytosis / macrophage activation syndrome

**DOI:** 10.1186/1546-0096-9-S1-P239

**Published:** 2011-09-14

**Authors:** Jan-Inge Henter, AnnaCarin Horne, Bo Magnusson, Stefan Hagelberg

**Affiliations:** 1 Childhood Cancer Research Unit; 2 Pediatric Rheumatology Unit; Department of WomenÂ´s and ChildrenÂ´s Health, Karolinska Institutet, Karolinska University Hospital Solna, Stockholm, Sweden

## Background/aim

Rheuma-associated hemophagocytic lymphohistiocytosis (Rh-HLH), also called macrophage activation syndrome (MAS), is a severe complication of systemic inflammatory disorders. Rh-HLH has clinical and laboratory similarities to other forms of HLH, and is potentially life threatening. Treatment of Rh-HLH has not been standardized yet, but it commonly includes a variety of agents such as high-dose corticosteroids, cyclosporine, intravenous immunoglobulin and, in severe cases, sometimes etoposide. Here we report on the experience of etoposide in two children with severe Rh-HLH admitted to the Karolinska Children’s Hospital over the 6 months July 2010 to Dec 2010.

## Methods

1) A 16-yr old boy with systemic lupus erythematosus was referred from the local hospital because of accelerating inflammatory disease. He was initially administered methyl-prednisolone (MP) pulses for 3 days followed by prednisolone 90 mg daily. He developed clinically signs of severe CNS-affection, confirmed by MRI, and diagnostic criteria consistent with MAS. Treatment with etoposide 75 mg/m2 weekly was administered for a month. His CNS symptoms rapidly improved, he recovered fully and a subsequent MRI was normal.

2) A 9-yr old girl with systemic juvenile idiopathic arthritis on treatment with tocilizumab and oral methotrexate was infected with EBV. She developed a fulminant picture of MAS. She was initially administered MP-pulses. However, within 24 hours her cerebral function deteriorated further, and therapy was intensified with etoposide 100 mg/m2 (a total of 8 courses), rituximab 375 mg/m2 and dexamethasone. She also developed seizures and an abnormal MRI. She recovered fully, and a subsequent MRI was normal.

## Results

Two children with Rh-HLH and CNS affection both responded well and without severe side effects to weekly etoposide 75-100 mg/m2 as a complement to high-dose MP pulses.

## Conclusion

Etoposide is worth considering in severe Rh-HLH.

